# EFFECTS OF MINDFULNESS ON COGNITION IN ADULTS WITH MILD COGNITIVE IMPAIRMENT: A META-ANALYSIS

**DOI:** 10.1093/geroni/igad104.2062

**Published:** 2023-12-21

**Authors:** Rose Lin, Jingjing Su, Kathi Heffner

**Affiliations:** University of Rochester, Rochester, New York, United States; Hong Kong Polytechnic University, Hong Kong, Hong Kong; University of Rochester Medical Center, Rochester, New York, United States

## Abstract

Mindfulness, or present-moment awareness, has been linked to greater psychological well-being and cognitive health in older adults. Recent studies on mindfulness-based intervention further explored its cognitive benefits in adults at the preclinical stage of dementia, yet showing mixed results. Therefore, this meta-analysis synthesizes the effects of mindfulness-based intervention on cognitive function for adults with mild cognitive impairment (MCI). Literature search was conducted in MEDLINE, EMBASE, PubMed, CINAHL, Web of Science, and Scopus. Randomized controlled trials that examined the effects of a mindfulness-based intervention on cognitive function in adults with MCI were included. Study effects were calculated by standard mean difference (SMD) and its 95% confidence interval using random effects model. Meta-regression was conducted to test the effects of methodological quality and intervention characteristics (qualification of interveners, types of mindfulness approach and control treatment) on trial effectiveness. Twelve trials involving 530 participants (female=64.26%, mean age=71.21 years) were included. Half of the reviewed studies were pilot trials with a mean attrition rate of 18.7%. Pooled evidence suggested that mindfulness-based intervention exerts a small-to-moderate effect on global cognition (SMD=0.40;95%CI[0.14,0.66]), visuospatial ability (SMD=0.25;95%CI[0.03,0.48]) and long-term memory (SMD=0.21; 95%CI[0.01,0.41]). No significant effect was detected for short-term memory, executive function, and attention. Meta-regression showed that none of the covariates moderate the treatment effect. This meta-analysis suggested that mindfulness-based intervention might improve cognitive function and its more specific domains in adults with MCI. More research with larger sample is warranted to confirm the effectiveness and examine the underlying mechanism to explain the improved outcomes.

